# Effectiveness of a Fluid-Collection Device for the Duodenoscope Biopsy Channel During Endoscopic Retrograde Cholangiopancreatography

**DOI:** 10.3390/medicina61122203

**Published:** 2025-12-12

**Authors:** Ho Seung Lee, Jae Min Lee, Inhwan Jung, Chaeyun Sung, Seokju Hong, Tae In Kim, Han Jo Jeon, Hyuk Soon Choi, Eun Sun Kim, Bora Keum, Yoon Tae Jeen, Hong Sik Lee

**Affiliations:** 1 Department of Internal Medicine, Korea University Anam Hospital, Seoul 02841, Republic of Korea; 2 College of Medicine, Korea University, Seoul 02841, Republic of Korea

**Keywords:** biopsy port, endoscopic devices, endoscopic retrograde cholangiopancreatography, leakage, procedure-related infections

## Abstract

*Background and Objectives*: Fluid leakage through the biopsy port during endoscopic retrograde cholangiopancreatography (ERCP) is a common procedural challenge that can compromise efficiency and increase the risk of contamination. In this study, we aimed to evaluate factors associated with fluid leakage and describe the performance of a novel leakage-collection device. *Materials and Methods*: A total of 183 patients who underwent ERCP between June and September 2024 at a single center were included. Fluid leakage was measured using a prototype collection device. Patients were categorized into low- (*n* = 126) and high-leakage (*n* = 57) groups based on the mean leakage volume. Logistic regression models were used to identify the clinical and procedural factors associated with high leakage. *Results*: Higher procedural complexity (Schutz grade) was strongly associated with high leakage (OR per grade increase, 3.66; 95% CI, 2.33–6.07; *p* < 0.001). In multivariable analysis, prolonged procedure duration (aOR, 1.11; 95% CI, 1.06–1.17) and more frequent duodenal flushing (aOR, 5.38; 95% CI, 1.80–18.75) were independently associated with high fluid leakage. Biliary plastic stenting (aOR, 4.53; 95% CI, 1.54–14.69) and malignancy (aOR, 3.02; 95% CI, 1.13–8.39) also showed significant associations. The device collected a mean fluid volume of 11 mL per procedure. *Conclusions*: Prolonged procedure duration and frequent duodenal flushing were key predictors of increased fluid leakage during ERCP. The leakage-collection device enabled measurement and containment of biopsy-port fluid but requires further validation to determine its broader clinical utility.

## 1. Introduction

Endoscopic retrograde cholangiopancreatography (ERCP) remains a cornerstone technique in the diagnosis and management of pancreaticobiliary diseases, despite major advancements in endosonographic-guided interventions [1,2,3,4,5]. Over the past decade, the volume of ERCP procedures, the role of ERCP as a therapeutic interventional tool, and case complexity have all increased significantly [6].

As procedural demands and device complexity have evolved, attention has turned to infection control and equipment design. Duodenoscopes used in ERCP feature larger instrument channels and an integrated elevator mechanism to enable precise accessory control. However, their meticulous disinfection procedures and concerns over infection outbreaks have prompted the development of duodenoscopes with disposable elevator caps [7,8,9,10].

During ERCP, repeated insertion and removal of accessories such as guidewires, retrieval baskets, biopsy forceps, or stents can exacerbate fluid leakage from the biopsy port. Such leakage is frequently encountered and may intermittently affect scope handling or contribute to minor environmental contamination in the endoscopy suite. Moreover, aerosolization of biofluids increases the risk of transmission to medical staff, particularly during airborne infectious disease outbreaks such as coronavirus disease 2019 (COVID-19) [11,12,13,14]. Although the biopsy port contains a valve to reduce backflow, leakage remains a commonly observed phenomenon. Notably, despite its clinical relevance, few studies have systematically examined the procedural or patient-level factors that contribute to fluid leakage during ERCP.

To address this gap, we used a novel leakage-collection device designed to reduce fluid spillage from the biopsy port during ERCP.

## 2. Materials and Methods

### 2.1. Patients and Data Collection

This single-center, retrospective study was conducted at the Korea University Anam Hospital between 1 June and 30 September 2024, using prospectively collected data. Adult patients (≥18 years) who underwent ERCP for standard clinical indications related to pancreatic or biliary diseases were eligible for inclusion. All procedures were performed by endoscopists with >10 years of experience in therapeutic endoscopy. Patients with altered gastrointestinal anatomy who underwent ERCP using a conventional gastroscope were excluded. All methods were performed in accordance with the relevant guidelines and regulations. Because of the retrospective nature of the study, the need to obtain the informed consent was waived by the Ethics Committee of Korea University Anam Hospital.

Baseline demographic and clinical data, including age, sex, and comorbidities, were recorded. Procedural characteristics were also documented, and the fluid volume was measured using a prototype leakage collection device to ensure correlation with specific ERCP techniques. Because the distribution of fluid leakage volume was highly right-skewed with a median value of 0 mL, patients were categorized into low- and high-leakage groups using the mean leakage volume (11.25 mL) as a pragmatic and data-driven cutoff. To evaluate the robustness of this cutoff, a sensitivity analysis was additionally performed using the 75th percentile (Q3) to define high leakage. Similarly, the frequency of duodenal irrigation using a 30 mL syringe was dichotomized based on the mean number of irrigations.

### 2.2. Device and Technique

**Figure 1:**
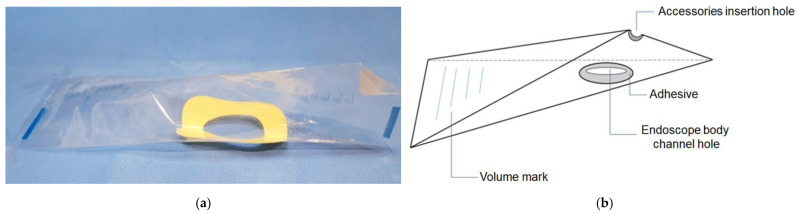
(**a**) Bile-collector device used to prevent fluid leakage during ERCP. (**b**) Schematic diagram illustrating the structural components of the bile-collector device.

**Figure 2:**
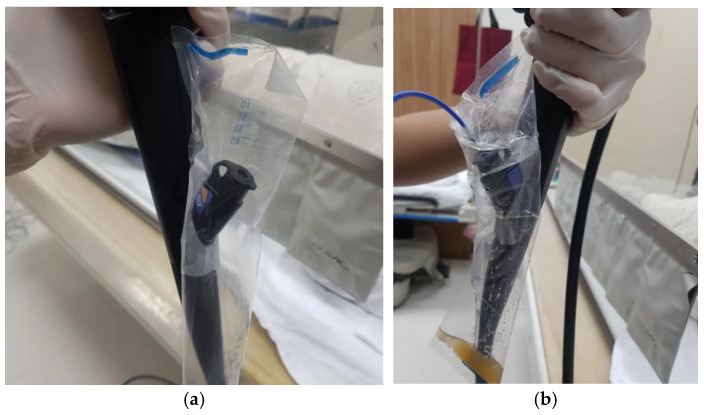
(**a**) Bile-collector device mounted on the duodenoscope prior to the procedure. (**b**) Collected fluid visible inside the device following completion of the procedure.

All ERCP procedures were performed under conscious sedation using intravenous propofol, and a duodenoscope (TJF-260 or JF-260, Olympus, Tokyo, Japan) was used. The design, structure, and schematic illustrations of the leakage-collection device (Lutecs Co., Ltd., Seoul, Republic of Korea) are shown in Figure 1. Figure 2a shows the bile collector mounted on the duodenoscope, and Figure 2b depicts the fluid collected within the device after the procedure.

During ERCP, the leakage-collection device was securely affixed to the biopsy port of the duodenoscope. The volume of collected fluid was measured at the end of each procedure. The leaked fluid observed during ERCP originated from the endoscope working channel and consisted of a heterogeneous mixture of materials encountered during the procedure. This included variable proportions of bile, trace amounts of contrast medium, fine stone debris, minute food particles, and other particulate or fluid components mixed with irrigation solution.

Duodenal irrigation was performed as needed when bile, debris, or foamy secretions obscured the visual field, as well as before and after cannulation and following stone extraction to ensure clear visualization of the ampulla. Guidewire-assisted cases were defined as procedures in which a guidewire was used at any stage, including during cannulation or accessory passage, acknowledging that guidewire usage varies among endoscopists. Procedure complexity was classified according to the Schutz grading system (Grades 1–4) [15].

### 2.3. Statistical Analysis

Continuous variables are expressed as mean ± standard deviation (SD) or median with interquartile range, whereas categorical variables are presented as frequencies and percentages. Group comparisons were performed using Welch’s *t*-test or the Wilcoxon rank-sum test for continuous variables, and the chi-square test or Fisher’s exact test for categorical variables Comparisons across the four Schutz grades were conducted using analysis of variance or the Kruskal–Wallis test, as appropriate. To evaluate the association between procedural complexity and fluid leakage, both ordinal modeling (Schutz grade treated as a continuous variable) and binary comparison (low-grade [1,2] vs. high-grade [3,4]) were performed using logistic regression. Univariate and multivariate logistic regression analyses were conducted to identify factors associated with high fluid leakage. Crude odds ratios (ORs) with 95% confidence intervals (CIs) were calculated in the univariate analysis. To avoid overfitting given the limited number of high-leakage events, the multivariable model was constructed using a parsimonious, clinically informed selection of covariates rather than including all variables with *p* < 0.10 in the univariable analysis. These selected variables were then entered into the multivariable model to estimate adjusted odds ratios (aORs). As a sensitivity analysis, logistic regression was repeated using an alternative definition of high leakage based on the 75th percentile (Q3) of fluid volume, to assess whether model findings were robust to different cutoff choices. Two-sided *p* < 0.05 was considered statistically significant. All analyses were conducted using the R software (version 4.4.3; R Foundation for Statistical Computing, Vienna, Austria).

## 3. Results

In total, 183 patients were included in this study (Table 1). The mean patient age was 69.3 ± 13.1 years, and 107 (58.5%) were male. The most common diagnosis was biliary stone disease (*n* = 108; 59.0%), followed by benign strictures (*n* = 22; 12.0%), bile duct or gallbladder cancer (*n* = 20; 10.9%), and pancreatic cancer (*n* = 10; 5.5%). Overall, 81 procedures (44.3%) were first-time ERCPs and 102 (55.7%) were repeat ERCPs in patients with a prior sphincterotomy.

The mean procedure duration was 16.1 ± 12.6 min, and duodenal irrigation with a 30 mL syringe was performed an average of 5.9 ± 2.4 times. The mean fluid leakage volume was 11.3 ± 15.5 mL. Several patients underwent more than one procedure, leading to overlapping use of endoscopic accessories. The most frequently used accessory was the stone extraction basket (*n* = 173, 94.5%), followed by the retrieval balloon catheter (*n* = 153, 83.6%) and guidewire-assisted techniques (*n* = 127, 69.4%). Biliary plastic stents were placed in 99 patients (54.1%), whereas biliary metal stents were used in only 9 (4.9%). Biopsy forceps and biliary stents were used in 22 (12.0%) and 81 (44.3%) patients, respectively. Among patients who underwent stone extraction, both a retrieval basket and an extraction balloon were used in 149 procedures.

### 3.1. Comparison Between Low-Leakage and High-Leakage Groups

Patients were stratified into low- (*n* = 126) and high-leakage (*n* = 57) groups based on the fluid leakage volume (Table 2). No significant differences were observed in mean age (69.3 ± 13.5 vs. 69.5 ± 12.5 years, *p* = 0.93) or sex distribution (71/126 [56.3%] vs. 36/57 [63.2%] male, *p* = 0.48). The diagnostic distribution differed significantly between groups. Biliary stone disease was more common in the low-leakage group (86/126 [68.0%] vs. 22/57 [38.6%]). Conversely, bile duct or gallbladder cancer was more frequent in the high-leakage group (14/57 [24.6%] vs. 6/126 [4.8%], *p* = 0.002).

Procedure duration was significantly longer in the high-leakage group (27.2 ± 13.7 vs. 11.1 ± 8.1 min, *p* < 0.001), with more frequent duodenal flushing (7.9 ± 2.1 vs. 5.0 ± 2.0 times, *p* < 0.001) than in the low-leakage group. The high-leakage group had a significantly higher mean fluid volume (30.5 ± 13.7 vs. 2.5 ± 4.3 mL, *p* < 0.001) than the low-leakage group.

The use of guidewires was markedly higher in the high-leakage group (96.5% vs. 57.2%, *p* < 0.001), while stone extraction baskets were used less frequently (84.2% vs. 99.2%, *p* < 0.001) than in the low-leakage group. Biopsy forceps were also more frequent in the high-leakage group (24.6% vs. 6.3%, *p* = 0.001). Biliary plastic stenting was significantly more frequent in the high-leakage group (87.7% vs. 38.9%, *p* < 0.001), whereas biliary stent removal was more common in the low-leakage group (52.4% vs. 26.3%, *p* = 0.002). No significant between-group differences were observed in the use of retrieval balloon catheters (*p* = 0.35) or biliary metal stents (*p* = 0.82).

### 3.2. Association Between Procedural Complexity (Schutz Grade) and Fluid Leakage

Fluid leakage increased significantly with procedural complexity as classified by the Schutz grading system (Table 3). The median leakage volume was 0 mL in Grade 1 and 2 procedures, whereas Grade 3 and 4 procedures showed markedly higher leakage (median 20 mL for both groups). A Kruskal–Wallis test demonstrated a significant difference in leakage volume across the four grades (χ^2^ = 59.6, *p* < 0.001).

In a logistic regression model, higher Schutz grades were strongly associated with high-volume leakage. When modeled as a continuous variable (Grade 1–4), the odds of high leakage increased 3.66-fold per one-grade increment (OR 3.66; 95% CI 2.33–6.07; *p* < 0.001). Dichotomizing the Schutz grade into low-grade (1–2) vs. high-grade (3–4) similarly showed a significant association (OR 12.47; 95% CI 5.71–29.07; *p* < 0.001) (Supplementary Table S1)

### 3.3. Association Between Procedural Factors and Fluid Leakage Volume

Associations between the individual procedures and leakage volume are summarized in Table 4. Biliary plastic stenting was associated with significantly higher leakage volume (17.9 ± 16.9 vs. 3.4 ± 8.5 mL, *p* < 0.001), whereas biliary stent removal was linked to lower leakage volumes (5.9 ± 11.1 vs. 15.5 ± 17.1 mL, *p* < 0.001). Biopsy forceps use resulted in more leakage (27.3 ± 22.3 vs. 9.1 ± 12.9 mL, *p* = 0.001), as did guidewire-assisted procedures (15.4 ± 16.2 vs. 1.8 ± 7.4 mL, *p* < 0.001). A dose-dependent trend was observed with guidewire use: leakage volumes increased with the number of insertions (0 insertions: 1.8 ± 7.4 mL; 1:10.9 ± 13.6 mL; 2:27.7 ± 16.8 mL; 3:30.0 mL). In contrast, biliary metal stent and retrieval balloon catheter use were not significantly associated with leakage volume (*p* = 0.15 and *p* = 0.47, respectively). Notably, patients in whom stone extraction baskets were used exhibited significantly lower leakage volumes than those in whom this was not used (10.4 ± 15.2 vs. 26.5 ± 12.5 mL, *p* = 0.003).

### 3.4. Predictive Factors for Significant Fluid Leakage in ERCP: Results from Logistic Regression Analysis

Table 5 presents the results of univariate and multivariate logistic regression analyses. In univariable analysis, several factors were significantly associated with high fluid leakage, including malignancy (OR, 4.65; 95% CI, 2.20–9.79, *p* < 0.001), longer procedure duration (OR, 1.14; 95% CI, 1.10–1.19; *p* < 0.001), frequent duodenal flushing (≥6 times) (OR, 20.07; 95% CI, 7.47–53.96; *p* < 0.001), and guidewire use (≥1) (OR, 20.63; 95% CI, 4.82–88.31; *p* < 0.001). Additionally, biliary plastic stenting (OR, 11.22; 95% CI, 4.71–26.75; *p* < 0.001) and the use of biopsy forceps (OR, 4.80; 95% CI, 1.88–12.25; *p* = 0.001) exhibited a strong association with increased fluid leakage. In contrast, stone extraction basket use (OR, 0.04; 95% CI, 0.01–0.35; *p* = 0.003) and biliary stent removal (OR, 0.33; 95% CI, 0.16–0.64; *p* = 0.001) were associated with significantly lower fluid leakage.

In the multivariable analysis, longer procedure duration (adjusted OR, 1.11; 95% CI, 1.06–1.17; *p* < 0.001) and more frequent duodenal flushing (adjusted OR, 5.38; 95% CI, 1.80–18.75; *p* = 0.004) were independently associated with higher odds of belonging to the high-leakage group. Biliary plastic stenting was also associated with increased leakage (adjusted OR, 4.53; 95% CI, 1.54–14.69; *p* = 0.008), and malignancy showed a significant association as well (adjusted OR, 3.02; 95% CI, 1.13–8.39; *p* = 0.029). Age and sex were not significantly associated with high leakage in the adjusted model. Other accessory-related variables—including guidewire use, stone extraction baskets, retrieval balloon catheters, and biopsy forceps—were not included in the final multivariable model because of collinearity with procedural complexity indicators and concerns about overfitting.

### 3.5. Sensitivity Analysis Using an Alternative Cutoff

As a sensitivity analysis, high leakage was redefined using the 75th percentile (Q3 = 20 mL). Based on this cutoff, 53 patients (29.0%) were classified into the high-leakage group, while 130 (71.0%) were categorized as low-leakage. The multivariable model using this alternative definition yielded similar findings: Malignancy (aOR 3.80; 95% CI, 1.41–10.76), longer procedure duration (aOR 1.12; 95% CI, 1.07–1.18), more frequent duodenal flushing (aOR 5.59; 95% CI, 1.74–22.01), and biliary plastic stenting (aOR 3.34; 95% CI, 1.10–11.10) remained significantly associated with high leakage. Age and sex were not significant predictors.

Detailed results are provided in Supplementary Table S2.

## 4. Discussion

This study aimed to quantify biopsy-port fluid leakage during ERCP and identify procedural factors associated with high-volume leakage. Leakage increased with procedural complexity, particularly with higher Schutz grades, longer procedure duration, and more frequent duodenal irrigation. The leakage collection device captured measurable volumes of fluid during the procedure and helped contain spillage around the biopsy port. Overall, leakage was predominantly influenced by procedural characteristics.

The increasing number and complexity of ERCP procedures have raised growing concerns about post-endoscopic infections, which may be underestimated [6,16,17]. Although outbreaks associated with multidrug-resistant organisms have drawn attention, the true incidence of ERCP-related infections remains uncertain [18,19,20,21,22]. The current estimates suggest infection risk after ERCP at approximately 0.8%, underscoring the need for improved preventive strategies [19]. Although interventions, such as disposable elevator caps, have been introduced [7], a comprehensive approach, including antimicrobial prophylaxis, optimized endoscope reprocessing, routine surveillance, and evidence-based infection control protocols, remains essential [14,19,22,23].

Biopsy port leakage during ERCP is not only a procedural inconvenience but also a potential source of environmental contamination and bioaerosol dispersion, particularly in the context of COVID-19 and other emerging infectious diseases [14,17,19,24,25]. Although biopsy port valves are designed to minimize backflow, they often fail to contain leakage entirely, necessitating additional measures to prevent contamination. In this study, we evaluated a novel leakage collection device that successfully collected an average of 11 mL of fluid per procedure. The device appeared to prevent visible fluid from reaching the floor in most cases. However, minor seepage at the attachment sites and accessory entry points was still observed, indicating that further refinements in the device design may improve the performance.

In our study, procedural complexity was strongly associated with fluid leakage. Higher Schutz grades—representing more technically demanding ERCPs—were associated with greater leakage volumes. This pattern may reflect prolonged instrument manipulation, more frequent accessory exchanges, and increased need for duodenal irrigation during complex procedures. This association was also quantitatively robust. Leakage volume increased progressively across Schutz grades, and the overall trend was highly significant (Table 3). In regression analyses, each one-grade increase in procedural complexity was associated with a 3.66-fold higher odd, and categorizing procedures as low-grade (1–2) versus high-grade (3–4) similarly demonstrated a strong association. These findings suggest that complex ERCPs may create procedural conditions that facilitate greater fluid escape. While the Schutz grade itself is not a direct causal factor, it likely functions as an aggregate indicator of procedural demands—such as repeated instrument exchanges, prolonged manipulation, and more frequent irrigation—that contribute to increased leakage.

Beyond procedural complexity, several procedural characteristics showed independent associations with higher fluid leakage. Longer procedure duration (adjusted OR, 1.11; 95% CI, 1.06–1.17; *p* < 0.001) and more frequent duodenal flushing (adjusted OR, 5.38; 95% CI, 1.80–18.75; *p* = 0.004) were significantly associated with high leakage in the multivariable analysis. These associations may reflect procedural situations requiring repeated visualization and extended instrument manipulation rather than indicating direct causal relationships. Biliary plastic stenting (adjusted OR, 4.53; 95% CI, 1.54–14.69; *p* = 0.008) and underlying malignancy (adjusted OR, 3.02; 95% CI, 1.13–8.39; *p* = 0.029) also demonstrated significant associations, suggesting that clinical scenarios involving more therapeutic steps or more advanced disease may coincide with increased leakage. Age and sex were not associated with high leakage after adjustment. Accessory-related variables—including guidewire use, retrieval balloon catheters, and biopsy forceps—showed associations in univariate analyses but were not included in the multivariable model because of strong collinearity with procedural complexity and concerns regarding overfitting. Their apparent univariate associations may therefore reflect the procedural contexts in which these tools are used rather than independent effects. Taken together, these findings indicate that fluid leakage during ERCP tends to occur under procedural conditions characterized by greater technical demands, more extensive handling, and repeated irrigation, rather than being uniformly distributed across cases.

In contrast, certain procedures, specifically the use of a stone extraction basket and biliary stent removal, were associated with lower fluid leakage volumes in the univariate analysis. Although these techniques tend to appear more frequently in cases with lower leakage, this pattern may simply reflect underlying procedural characteristics—such as shorter procedure duration or fewer accessory exchanges—rather than a direct protective effect. Because of concerns about overfitting in the multivariable model, these variables were not evaluated for independent associations. Stone extraction baskets are frequently used in straightforward choledocholithiasis cases where the procedures are relatively simple and require fewer accessory manipulations and minimal duodenal flushing. Similarly, biliary stent removal may involve fewer procedural steps and less extensive accessory exchanges than stent placement, which may be one reason why these procedures tend to show lower leakage. Collectively, these findings suggest that fluid leakage is closely related to procedural complexity: more intricate interventions tend to be accompanied by higher leakage, whereas simpler and shorter procedures are generally associated with lower leakage.

This study has some limitations. First, it was conducted at a single center, which potentially limits its generalizability. Second, although all procedures were performed by experienced endoscopists, operator-dependent variability may still exist. Third, although accessory use was documented, the duration of use was not measured, which could further clarify its association with leakage. Fourth, environmental contamination and procedural efficiency were not directly measured; therefore, any potential benefit of the leakage-collection device in reducing contamination or improving workflow cannot be confirmed from this study. Finally, this study focused primarily on procedural factors; therefore, future research should investigate the potential associations between patient-specific factors, such as duodenal motility or sphincter of Oddi function. Furthermore, biliary stent removal is routinely performed under ERCP at our center to confirm ductal clearance, which may have increased the proportion of follow-up ERCPs and introduced potential selection bias. Although high leakage was initially defined using a mean-based cutoff, a sensitivity analysis using the 75th percentile yielded similar associations, suggesting that the main findings were robust to alternative threshold definitions.

## 5. Conclusions

The leakage-collection device captured a measurable amount of fluid from the biopsy port; however, further studies are needed to determine its impact on contamination reduction or procedural safety.

## Figures and Tables

**Table 1:** Baseline characteristics of patients and procedural details (n= 183).

Variable	Total (*n* = 183)
Age, years (mean ± SD)	69.3 ± 13.1
Male, n (%)	107 (58.5)
Diagnosis, n (%)	
Biliary stone disease	108 (59)
IgG4-related cholangitis	3 (1.6)
Benign stricture	22 (12)
Benign tumor	2 (1.1)
Bile duct or gallbladder cancer	20 (10.9)
Pancreatic cancer	10 (5.5)
Bile leak	8 (4.4)
Others	10 (5.5)
Procedure time, min (mean ± SD)	16.1 ± 12.6
Duodenal irrigation frequency, times (mean ± SD)	5.9 ± 2.4
Volume of leaked fluid, mL (mean ± SD)	11.3 ± 15.5
ERCP status	First 81 (44.3%), Repeat 102 (55.7%)
Endoscopic procedure, n (%)	
Guidewire-assisted	127 (69.4)
Stone extraction basket	173 (94.5)
Retrieval balloon catheter	153 (83.6)
Biopsy forceps	22 (12)
Biliary metal stenting	9 (4.9)
Biliary plastic stenting	99 (54.1)
Biliary stent removal	81 (44.3)

Values are presented as mean ± standard deviation or number (percentage), as appropriate. Multiple procedures performed per patient may have resulted in overlapping counts. Others include lymphoma (*n* = 3), advanced gastric cancer (*n* = 1), rectal cancer (*n* = 1), hepatocellular carcinoma (*n* = 3), and ampulla of Vater cancer (*n* = 2).

**Table 2:** Comparison of clinical and procedural characteristics between low- and high-leakage groups.

Variable	Low-Leakage Group (*n* = 126)	High-Leakage Group (*n* = 57)	*p*-Value
Age, years (mean ± SD)	69.3 ± 13.5	69.5 ± 12.5	0.93
Male, n (%)	71 (56.3)	36 (63.2)	0.48
Diagnosis, n (%)			0.002
Biliary stone disease	86 (68)	22 (39)	
IgG4-related cholangitis	3 (2.4)	0	
Benign stricture	13 (10)	9 (16)	
Benign tumor	1 (0.8)	1 (1.8)	
Bile duct or gallbladder cancer	6 (4.8)	14 (25)	
Pancreatic cancer	5 (4.0)	5 (8.8)	
Bile leak	7 (5.6)	1 (1.8)	
Others	5 (4.0)	5 (8.8)	
Procedure time, min (mean ± SD)	11.1 ± 8.1	27.2 ± 13.7	<0.001
Duodenal flushing, times (mean ± SD)	5.0 ± 2.0	7.9 ± 2.1	<0.001
Volume of leaked fluid, mL (mean ± SD)	2.5 ± 4.3	30.5 ± 13.7	<0.001
Endoscopic procedure, n (%)			
Guidewire-assisted procedure	72 (57.2)	55 (96.5)	<0.001
Stone extraction basket	125 (99.2)	48 (84.2)	<0.001
Retrieval balloon catheter	108 (85.7)	45 (78.9)	0.35
Biopsy forceps	8 (6.3)	14 (24.6)	0.001
Biliary metal stenting	7 (5.6)	2 (3.5)	0.82
Biliary plastic stenting	49 (38.9)	50 (87.7)	<0.001
Biliary stent removal	66 (52.4)	15 (26.3)	0.002

Values are presented as mean ± standard deviation or number (percentage), unless otherwise indicated. *p*-values were calculated using the *t*-test, chi-square test, or Fisher’s exact test, as appropriate. “Others” included the following diagnoses: Low-leakage group: lymphoma (*n* = 3), hepatocellular carcinoma (*n* = 2); High-leakage group: hepatocellular carcinoma (*n* = 1), rectal cancer (*n* = 1); ampulla of Vater cancer (*n* = 2), advanced gastric cancer (*n* = 1).

**Table 3:** Fluid leakage according to Schutz grade.

Schutz Grade	n	Mean Leak (mL)	SD	Median Leak (mL)	IQR
Grade 1	59	5.1	10.8	0	0–5
Grade 2	82	7.8	11.7	0	0–10
Grade 3	34	27.9	18.7	20	11.2–40
Grade 4	8	21.2	9.9	20	17.5–22.5

**Table 4:** Comparison of fluid leakage volume according to procedural factors in ERCP.

Procedure	Category	n	Leaked Fluid (mL)	*p*-Value
Biliary metal stenting	No	174	11.5 ± 15.7	0.15
	Yes	9	6.7 ± 8.7	
Biliary plastic stenting	No	84	3.4 ± 8.5	<0.001
	Yes	99	17.9 ± 16.9	
Biliary stent removal	No	102	15.5 ± 17.1	<0.001
	Yes	81	5.9 ± 11.1	
Biopsy forceps	No	161	9.1 ± 12.9	0.001
	Yes	22	27.3 ± 22.3	
Guidewire-assisted procedure	No	56	1.8 ± 7.4	<0.001
	Yes	127	15.4 ± 16.2	
	3 times	1	30 ± NA	
	2 times	33	27.7 ± 16.8	
	1 time	93	10.9 ± 13.6	
	0 times	56	1.8 ± 7.4	
Retrieval balloon catheter	No	30	13.3 ± 17.5	0.47
	Yes	153	10.8 ± 15.1	
Stone extraction basket	No	10	26.5 ± 12.5	0.003
	Yes	173	10.4 ± 15.2	

ERCP, endoscopic retrograde cholangiopancreatography. Values are presented as mean ± standard deviation. *p*-values were calculated using the *t*-test or ANOVA, where applicable.

**Table 5:** Predictive factors associated with high fluid leakage during ERCP: logistic regression analysis.

Univariate Analysis			Multivariate Analysis	
Variable	OR (95% CI)	*p*-Value	Adjusted OR (95% CI)	*p*-Value
Male	1.33 (0.70–2.53)	0.39	0.42 (0.16–1.05)	0.070
Age	1.00 (0.98–1.03)	0.93	0.98 (0.95–1.02)	0.318
Malignancy	4.65 (2.20–9.79)	<0.001	3.02 (1.13–8.39)	0.029
Procedure time (min)	1.14 (1.10–1.19)	<0.001	1.11 (1.06–1.17)	<0.001
Duodenal flushing (≥6 times)	20.07 (7.47–53.96)	<0.001	5.38 (1.80–18.75)	0.004
Guidewire use (≥1 time)	20.63 (4.82–88.31)	<0.001		
Guidewire use (≥2 times)	13.28 (5.48–32.16)	<0.001		
Stone extraction basket use	0.04 (0.01–0.35)	0.003		
Retrieval balloon catheter use	0.63 (0.28–1.40)	0.26		
Biopsy forceps use	4.80 (1.88–12.25)	0.001		
Biliary plastic stenting	11.22 (4.71–26.75)	<0.001	4.53 (1.54–14.69)	0.008
Biliary metal stenting	0.62 (0.12–3.07)	0.56		
Biliary stent removal	0.33 (0.16–0.64)	0.001		

Univariate and multivariate logistic regression analyses were performed. ERCP, endoscopic retrograde cholangiopancreatography; CI, confidence interval; OR, odds ratio; aOR, adjusted odds ratio.

## Data Availability

All data supporting the findings of this study are available from the corresponding author upon reasonable request.

## Supplementary Materials

The following supporting information can be downloaded at: https://www.mdpi.com/article/10.3390/medicina61122203/s1, Table S1: Association between Schutz grade and high fluid leakage, Table S2: Multivariable logistic regression using Q3-based high-leakage definition (sensitivity analysis).

## Abbreviation

ERCP: endoscopic retrograde cholangiopancreatography
