# Supplementary material for: Effectiveness of a Fluid-Collection Device for the Duodenoscope Biopsy Channel During Endoscopic Retrograde Cholangiopancreatography
Source: Medicina (Kaunas). 2025 Dec 12;61(12):2203. doi: 10.3390/medicina61122203 (PMC12734413; doi:10.3390/medicina61122203)
Supplement: Supplementary file 1 [file medicina-61-02203-s001.zip › medicina-3998767-supplementary.pdf]

Supplementary Table S1. Association between Schutz grade and high fluid leakage

**Variable**	**OR**	**95% CI**	**p-value**
Schutz grade (continuous)	3.66	2.33–6.07	<0.001
High-grade (3–4) vs Low-grade (1–2)	12.47	5.71–29.07	<0.001

Supplementary Table S2. Multivariable logistic regression using Q3-based high-leakage definition (sensitivity analysis)

Variable	aOR	95% CI	p-value
**Malignancy**	3.80	1.41–10.76	0.009
**Procedure time (min)**	1.12	1.07–1.18	<0.001
**Duodenal flushing (≥ 6 times)**	5.59	1.74–22.01	0.007
**Biliary plastic stent**	3.34	1.10–11.10	0.038
**Male**	0.52	0.19–1.34	0.185
**Age**	0.98	0.94–1.01	0.203
